# Immunogenic cell death inducers as anticancer agents

**DOI:** 10.18632/oncotarget.2266

**Published:** 2014-07-26

**Authors:** Oliver Kepp, Laura Senovilla, Guido Kroemer

**Affiliations:** Equipe 11 labellisée par la Ligue Nationale contre le cancer, INSERM U1138, Centre de Recherche des Cordeliers, Paris, France

It has been widely thought that the occasional success of anticancer chemotherapies is mediated by direct, efficient cytostatic or (better) cytotoxic effects of the antineoplastic agent on tumor cells. Nonetheless, there is accumulating evidence for the hypothesis that long-term clinical success (which is measured in years and decades rather than weeks and months) involves anticancer immune responses that are often mediated by T lymphocytes recognizing tumor-specific antigens. During recent years, a whole catalogue of mechanisms through which chemotherapeutics can stimulate immune responses has emerged. Thus, some therapeutic agents can stimulate immune effector cells either directly or – more frequently – by subverting the immunosuppressive circuitries that block antitumor immune responses [1]. In addition, some chemotherapeutic agents provoke immunogenic cancer cell death (ICD), meaning that they induce tumor cell death in a way that those cells elicit a specific immune response. ICD is characterized by a series of alterations that usually do not occur in the context of apoptosis: (i) the pre-apoptotic exposure of calreticulin (CRT) on the cell surface, (ii) release of ATP during the blebbing phase of apoptosis, and (iii) post-apoptotic exodus of the chromatin-binding protein high mobility group B1 (HMGB1). CRT exposure critically depends on a premortem endoplasmic reticulum stress response, ATP release on premortem autophagy, and HMGB1 exodus on secondary necrosis. CRT, ATP and HMGB1 bind to three receptor types (CD91 receptor, purinergic P2Y2 or P2X7 receptors, and toll-like receptor 4, respectively) that are present on dendritic cells or their precursors. CD91, P2Y2, P2RX7 and TLR4 promote engulfment of dying cells, attraction of dendritic cells into the tumor bed, production of interleukin-1β and presentation of tumor antigens, respectively [2].

Since (some of) the molecular characteristics of ICD are well studied, it has been possible to screen compound libraries for the presence of ICD inducers, which would cause (i) CRT exposure, (ii) ATP release and (iii) HMGB1 exodus in cultured human cancer cells [3]. Agents that induce the hallmarks of ICD in vitro could be validated by in vivo experiments using two complementary assays. First, it was possible to test the capacity of candidate ICD inducers to kill mouse cancer cell lines in vitro so that the resulting dead-cell preparation would elicit protective anticancer immune responses upon its subcutaneous injection into immunocompetent, syngenic mice. Second, the anticancer effects of ICD inducers on established tumors were found to be more efficient if such tumors evolved in immunocompetent (as opposed to immunodeficient) mice [2].

Using a combined in vitro screening assay, followed by in vivo validation experiments, we screened three chemical libraries (Table 1): (i) a collection of FDA-approved anticancer agents, (ii) the sum of all other FDA-approved molecules, and (iii) a series of 879 anticancer agents that constitute the “mechanistic diversity set” of the National Cancer Institute (NCI). This latter collection is composed by candidate drugs that have been selected based on their preclinical activity, mostly on human cancer cell lines, either in vitro or in vivo, in xenografted (immunodeficent) mice. Using a similar cutoff for distinguishing in vitro ICD inducers from agents that fail to induced ICD for all these chemical libraries, we observed that 7 among 114 approved anticancer agents could elicit the hallmarks of ICD in vitro [3], and we validated the capacity to stimulate ICD in vivo for 6 of those components [3, 4]. Among the 1040 FDA-approved drugs with indications different from antineoplastic, only a few components (which all were cardiac glycosides) were found to induce ICD [3, 5], meaning that – as expected – the frequency of ICD inducers is higher among anticancer agents than among the remaining pharmaceutical specialties. Importantly, among the NCI mechanistic diversity set, only 12 among 879 components were able to elicit the characteristics of ICD in vitro and only one agent withstood the rigors of in vivo validation [6].

**Table 1:** Identification of ICD inducers in distinct drug collections

Collection of drugs	Number of agents scored as ICD inducers *in vitro/*total (%)	Number of *in vivo* validated agents/total (%)	Validated ICD inducers
FDA approved anticancer drugs	7/114 (6.1%)	6/114 (5.3%)	Daunorubicin Docetaxel Doxorubicin Mitoxanthrone Oxaliplatin Paclitaxel
All other FDA approved drugs	10/1040 (1.0%)*	2/1040 (0.2%)*	Digitoxin Digoxin
NCI mechanistic set	12/879 (1.4%)*	1/879 (0.1%)*	Sptacidin

* Values significantly (p<0.01) lower than for FDA-approved anticancer drugs (Chi square analysis).

Although the methods that lead to the identification of ICD inducers can be criticized (and actually may fail to identify ICD inducers) [7, 8], these results support the contention that FDA-approved anticancer agents have a higher chance to elicit ICD than the drug candidates from the NCI (see Table 1 for statistical analyses). How can this difference be explained?

While FDA-approved drugs have passed the selection process of clinical evaluation, agents contained in the NCI panel are merely characterized for their preclinical ability to directly interfere with human cancer cell growth. We surmise here that clinical trials leading to FDA approval (as well as the subsequent clinical evaluation leading to the discontinuation of inefficacious therapies) has created an intrinsic bias in which those drugs that stimulate anticancer immune responses and thus superior efficiency have been selected for. If this contention would be correct, it will be important to shift the selection of immunostimulatory drugs from the clinical to the preclinical stage, obviously by means of their precocious immunological evaluation. This could be achieved by testing drugs for the induction of ICD hallmarks in human cancer cell lines, by their evaluation on cocultures of human cancer cells and leukocyte subpopulations, as well as by their preclinical testing on mouse tumors developing in immunocompetent mice, or preferentially on human cancers evolving on “humanized” rodents, i.e. mice that have been engineered to carry a human immune system.
